# Effectiveness of a physiotherapist-led triage and treatment service on WAITing time for adults with musculoskeletal pain referred to Australian public hospital physiotherapy clinics: a protocol for the WAIT-less trial

**DOI:** 10.1136/bmjopen-2024-091293

**Published:** 2025-01-15

**Authors:** Joshua M Hutton, Andrew R Gamble, Chris G Maher, Tarcisio F de Campos, Christopher S Han, Danielle Coombs, Mark Halliday, Lisa A Harvey, Nadine E Foster, Gustavo Machado, David Anderson, Laurent Billot, Bethan Richards, Michael Swain, Marnee McKay, Chris Needs, Jackie Chu, Timothy Shaw, Tom Lung, Ian A Harris, Joshua R Zadro, WAIT-less TRIAL INVESTIGATORS, Lisa Gridley

**Affiliations:** 1 Institute for Musculoskeletal Health, Sydney Local Health District, Sydney, New South Wales, Australia; 2 Sydney School of Public Health, Faculty of Medicine and Health, The University of Sydney, Sydney, New South Wales, Australia; 3 Royal Prince Alfred Hospital, Sydney Local Health District, Sydney, New South Wales, Australia; 4 Faculty of Medicine, Health and Human Sciences, Macquarie University, Sydney, New South Wales, Australia; 5 Concord Hospital, Sydney Local Health District, Sydney, New South Wales, Australia; 6 John Walsh Centre for Rehabilitation Research, Northern Sydney Local Health District, The University of Sydney, Kolling Institute, Sydney, New South Wales, Australia; 7 Kolling Institute, Faculty of Medicine & Health, The University of Sydney, Sydney, New South Wales, Australia; 8 STARS Education and Research Alliance, Surgical Treatment and Rehabilitation Service, The University of Queensland and Metro North Health, Brisbane, Queensland, Australia; 9 Faculty of Medicine and Health, School of Health Sciences, The University of Sydney, Sydney, New South Wales, Australia; 10 Sydney Musculoskeletal Health, Faculty of Medicine and Health, The University of Sydney, Sydney, New South Wales, Australia; 11 The George Institute for Global Health, University of New South Wales, Sydney, New South Wales, Australia; 12 Faculty of Medicine and Health, School of Medical Sciences, The University of Sydney, Sydney, New South Wales, Australia; 13 Faculty of Medicine & Health, School of Clinical Medicine, University of New South Wales, Sydney, New South Wales, Australia

**Keywords:** Physical Therapy Modalities, REHABILITATION MEDICINE, Waiting lists, Musculoskeletal disorders, Patient Care Management

## Abstract

**Introduction:**

Musculoskeletal pain is the second leading cause of disease burden in Australia, and there is a need to investigate new models of care to cope with the increasing demand for health services. This paper describes the protocol for a randomised controlled trial investigating whether a physiotherapist-led triage and treatment service is non-inferior for improving function at 6 months and superior for reducing treatment waiting times, compared with usual care for patients with musculoskeletal pain referred to public hospital outpatient physiotherapy clinics.

**Methods and analysis:**

A total of 368 participants (184 per arm) will be recruited from six public hospitals located in metropolitan Sydney, Australia. We will recruit adult patients newly referred to a public hospital physiotherapy outpatient clinic with a musculoskeletal condition that is typically managed by a physiotherapist (eg, back or neck pain, osteoarthritis, rehabilitation postorthopaedic surgery and sporting injuries). Participants will be randomised 1:1 to the physiotherapist-led triage and treatment service or usual care. A physiotherapist will telephone participants in the intervention group and match them to different modes and types of care based on baseline characteristics. Participants at low risk of poor outcomes (assessed by the Keele STarT MSK tool) will be given simple advice and education during this call and instructed to call back if their symptoms do not improve in 6 weeks. Participants at medium risk of poor outcomes or requiring postoperative rehabilitation will be offered a course of telehealth (videoconference) physiotherapy targeting exercise-based self-management. Participants at high risk of poor outcomes and/or with potential nonprogressive radiculopathy will be offered a course of usual clinic-based physiotherapy as per all participants in the usual care group. Physical function (Patient Specific Functional Scale) at 6 months postrandomisation and waiting time (time from randomisation to first treatment) are coprimary outcomes. Secondary outcomes include other patient outcomes (eg, pain), health resource use, adverse events, process measures (eg, adherence) and costs.

**Ethics and dissemination:**

This trial has approval from the Sydney Local Health District Human Research Ethics Committee (RPAH Zone; X24-0090 and 2024/ETH00585). Recruitment will commence in September 2024 and is expected to be completed by December 2025 with follow-ups completed by December 2026. The results of the trial will be submitted for publication in reputable international journals and presented at relevant national and international conferences.

**Trial registration number:**

ANZCTR (ACTRN12624000947505).

**STRENGTH AND LIMITATIONS OF THIS STUDY**

- This is a multicentre, two-arm, parallel-group randomised controlled trial with nested process and economic evaluation.
- Findings may provide information on the implementation of a physiotherapist-led triage and treatment service that could improve access to care within outpatient hospital physiotherapy departments.
- Participants will be blinded to the study hypothesis.
- Physiotherapists delivering the interventions will not be blinded.
- All study sites will be in metropolitan Sydney, so the findings might not be applied to rural and remote parts of Australia or other countries.

## Introduction

 Musculoskeletal pain is the second leading cause of disease burden in Australia, and there is a need to investigate new models of care to cope with the increasing demand placed on health services.^1 2^ Many Australians seeking care for musculoskeletal pain do not have private health insurance^3^ and join increasingly long waiting lists for clinic-based physiotherapy in publicly funded hospitals (where there is no cost to the patient). Long waiting times of up to 12 months^4^ for physiotherapy in public hospitals likely delay recovery for some patients with musculoskeletal pain and lead some to develop persistent disabling symptoms that are costly and complex to manage.^5^,^7^ A potential solution to reduce treatment waiting times is a model of care that can identify patients with simple musculoskeletal problems that can be managed with less resources (eg, brief telephone appointments and App-based home exercise programmes), thereby freeing up clinic-based resources for patients with more complex presentations.

A promising model of care that reduced treatment waiting times without compromising clinical outcomes in a large randomised controlled trial (RCT; n=2256) was the UK PhysioDirect telephone assessment and advice service for patients with musculoskeletal pain.^8^ PhysioDirect used an innovative clinical algorithm that supported physiotherapists to triage patients who needed clinic-based care while managing patients they judged to be able to self-manage via telephone consultation (exercise advice and advice leaflets).^8^ PhysioDirect reduced treatment waiting times compared with usual clinic-based care (median of 7 days vs 34 days, arm time ratio of 0.32, 95% CI 0.29 to 0.35) and was deemed equally clinically effective and seemingly safe.^8^ It was also acceptable to patients and physiotherapists in the UK.^8^

The PhysioDirect model of care inspired us to develop and evaluate a similar model of care to tackle the long waiting times in many outpatient physiotherapy clinics in Australia. Our initial ‘triage and treatment service’ was developed for a multidisciplinary rheumatology and physiotherapy clinic where it was later evaluated in a pilot and feasibility RCT for people with low back pain.^9^ In this trial, a rheumatologist called participants in the intervention group soon after they joined the clinic waiting list and matched them to different modes and types of care based on a baseline screening assessment. Participants at low risk of poor outcomes (assessed by the Keele STarT MSK tool)^10^ were given simple advice and education during this call and instructed to call back if their symptoms did not improve in 6 weeks. Participants at medium and high risk of poor outcomes were offered a course of telehealth (videoconference) physiotherapy targeting exercise-based self-management. High-risk participants were additionally encouraged to complete an online self-directed pain education programme. Participants with potential nonprogressive radiculopathy were offered a clinic-based rheumatology appointment with the option of clinic-based physiotherapy. Findings from the pilot and feasibility RCT and additional qualitative interviews were used to refine our new model of care and adapt it for use in outpatient physiotherapy clinics (‘physiotherapist-led triage and treatment service’) that manage a range of musculoskeletal conditions. A more detailed description of the development and refinement of our physiotherapist-led triage and treatment service is described elsewhere.^11^

This paper describes the protocol for an RCT (WAIT-less trial) investigating whether our new physiotherapist-led triage and treatment service is noninferior for improving function at 6 months and superior for reducing treatment waiting times (coprimary outcomes) compared with usual clinic-based physiotherapy care for patients with musculoskeletal pain referred to public hospital outpatient physiotherapy clinics.

## Methods and analysis

### Design

The WAIT-less trial is a pragmatic multicentre, two-arm, parallel-group RCT with nested process and economic evaluation. This protocol follows the Standard Protocol Items: Recommendations for Interventional Trials statement (https://www.spirit-statement.org/). The trial is registered with the Australian and New Zealand Clinical Trials Registry—trial registration number ACTRN12624000947505.

### Setting

The WAIT-less trial will be conducted in several public hospital outpatient physiotherapy clinics across Sydney Local Health District (SLHD) and South Eastern Sydney Local Health District (SESLHD) in NSW, Australia, and compare the physiotherapist-led triage and treatment service with usual clinic-based care. The participating hospitals provide diversity with respect to patient demographic characteristics, ethnicity, hospital setting and outpatient clinic staffing models. We will randomise, across all sites, 368 participants to this trial (ie, 184 per arm). Participants in the physiotherapist-led triage and treatment service group and usual care group will be treated at the hospital they are referred to.

### Eligibility criteria

**Figure 1:**
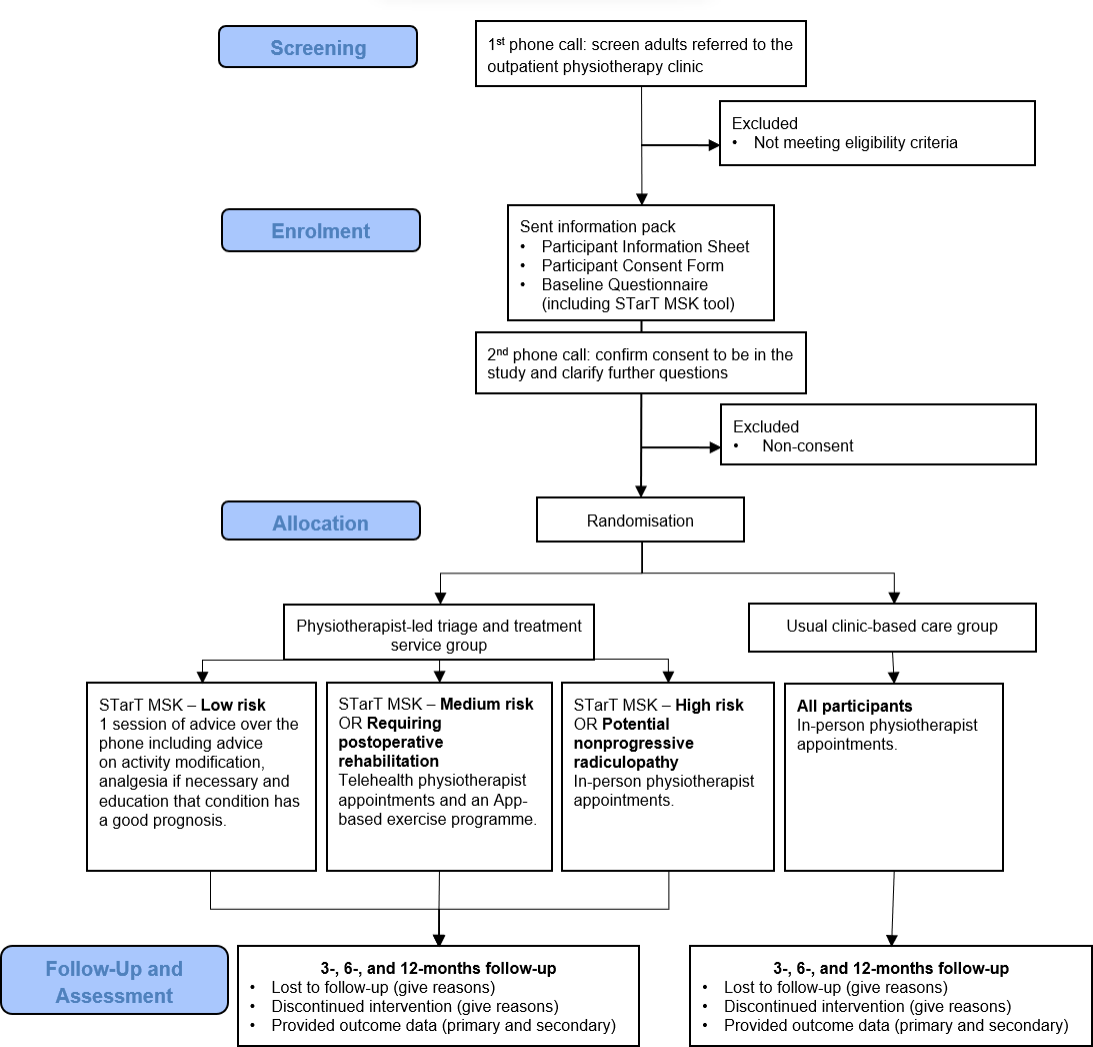
Trial design.

Patients referred to participating outpatient physiotherapy clinics are referred from Hospital Emergency Departments, inpatient units, outpatient hospital clinics and local General Practices. Occasionally, patients can self-refer but only at some hospitals and in extenuating circumstances. Potentially eligible newly referred patients will be contacted by a screening physiotherapist from either an SLHD or SESLHD site to determine if the patient is eligible and interested in participating in the trial. Figure 1 illustrates the trial design. Box 1 below describes the eligibility criteria for the trial.

Box 1 **Eligibility criteria**

**Inclusion criteria**

- Adult patient (≥18 years)
- New referral (defined as being referred within the last 6 weeks) to a public hospital physiotherapy outpatient clinic with a musculoskeletal condition or musculoskeletal pain (hereafter referred to as ‘musculoskeletal pain’) that would usually be managed by a physiotherapist. Examples include but are not limited to:
  - Back/neck pain
  - Osteoarthritis
  - Whiplash-associated disorders
  - Ankle sprains
  - Postfracture
  - Sporting injury (eg, ankle sprains)
  - Postorthopaedic surgery (eg, hip or knee replacement, rotator cuff repair)
- Willing to participate and provide follow-up data
- Can speak and read English to provide informed consent

**Exclusion criteria**

- Suspected serious underlying pathology or musculoskeletal conditions requiring urgent medical care (eg, malignancy, fracture, infection, inflammatory arthritis and joint dislocation)

- New referral strongly suggestive of concerning neurological features (eg, progressive radiculopathy or upper motor neuron lesion)
- Is on a postoperative exercise regimen prescribed by a surgeon which specifies the mode of care delivery (eg, needs to be provided in a clinic supervised by a physiotherapist)

- Requiring mobility progression or assistance weaning from a walking aid(s) whereby the person is at an increased fall risk and needs to be seen in the clinic
- Pregnant women.

### Participant recruitment

Initial contact with patients referred to the outpatient physiotherapy clinic will be made as soon as possible after a referral is received by a screening physiotherapist via telephone. During this call, patients will be invited to participate in the trial. If patients demonstrate interest, the screening physiotherapist will explain the trial and determine their eligibility using a screening form that contains the eligibility criteria. For all patients interested and eligible to be involved, the screening physiotherapist will send an email, SMS or mail out of the trial information pack that includes the Participant Information Statement Form (online supplemental appendix 1), Participant Consent Form (PCF—online supplemental appendix 2) and the baseline assessment questionnaire. Participants will be made aware that participation is voluntary, and they are free to withdraw at any time with no repercussions. Participants who receive the information pack via email or SMS will provide consent by ‘checking’ a radio button in an online survey through Research Electronic Data Capture (REDCap) and then proceed to the baseline assessment questionnaire online. For all interested and eligible patients who are not able to complete the PCF and baseline assessment questionnaire via SMS or email, they will be sent the trial information pack via post. Patients who receive the trial information pack via post will be provided with a pen and a postage-paid return envelope, so they can post their signed PCF and completed baseline assessment questionnaire to the screening physiotherapist.

The screening physiotherapist will offer all participants a telephone call if they need assistance completing the PCF or baseline assessment questionnaire. Participant consent will be documented in the REDCap trial database and the participant’s hospital medical record. This is as per normal practice and a medico-legal requirement. All patients deciding not to be involved in the trial will receive care from a health practitioner as per the standard of care in the clinic they were referred to. At any time during the study if the treating physiotherapist determines serious pathology must be excluded (suspicion of red flag pathology via assessment and screening questions), the participant will be referred to their general practitioner or referring doctor (eg, orthopaedic surgeon, rheumatologist or within hospital specialty) to obtain medical clearance before resuming physiotherapy as per best practice.

### Randomisation procedure

The 1:1 random allocation sequence will be independently generated in Stata by the team’s biostatistician and uploaded to REDCap. Allocation will be concealed as the biostatistician generating the sequence will not be involved in the recruitment of participants and the screening physiotherapist will not know the participant’s group allocation until their baseline data are entered into REDCap. The allocation sequence will also be concealed from potential participants and all on-site staff associated with the trial. The randomisation sequence will use randomly permuted blocks of variable sizes (2 and 4) to ensure equal numbers in both groups. Stratification variables for the randomisation sequence include trial sites and the three treatment subgroups. This will ensure that the intervention and control groups have a similar proportion of participants across different trial sites. The screening physiotherapist will telephone participants who consent (via completing the PCF—online supplemental appendix 2) and complete their baseline assessment to inform them of their group allocation. Table 1 describes the physiotherapist-led triage and treatment service and usual care group.

**Table 1:** Physiotherapist-led triage and treatment service and usual care protocol.

Treatment group and subgroup	Intervention protocol
Physiotherapist-led triage and treatment service	
*All participants*	For participants randomised to the physiotherapist-led triage and treatment service, the screening physiotherapist will match them to different modes and types of care during the call informing participants of their group allocation.
*Low risk* of poor outcomes	Participants at low risk of poor outcomes (Keele STarT MSK tool score 0–4)^28^ will be offered brief advice and education (via one telephone call with a physiotherapist) including advice on activity modification, analgesia if necessary and education that their condition has a good prognosis. This advice and education will be provided during the phone call where participants are informed of their trial arm allocation. Participants in this treatment subgroup will be asked to call the screening physiotherapist back if their symptoms have not improved in 6 weeks. If nil contact is received after 6 weeks, participants will be discharged from the physiotherapy service.
*Medium risk* of poor outcomes or requiring postoperative rehabilitation	Participants at medium risk of poor outcomes (Keele STarT MSK tool score 5–8) or requiring postoperative rehabilitation will be offered physiotherapy via telehealth. An appointment with a telehealth physiotherapist will be organised by the screening physiotherapist during the phone call where participants are informed of their trial arm allocation. Telehealth physiotherapy will consist of assessment, advice, education to support self-management and a tailored home-exercise programme delivered via the PhysioTherapy Exercise App (freely available online exercise prescribing software created by some of the study authors: www.physiotherapyexercises.com). The number of appointments offered will be at the discretion of the treating physiotherapist.
*High risk* of poor outcomes and/or with potential nonprogressive radiculopathy	Participants at high risk of poor outcomes (Keele STarT MSK tool score 9–12) and/or with potential nonprogressive radiculopathy will be offered a course of clinic-based (in-person) physiotherapy as is usually provided at the participating public hospital clinics. An appointment with a clinic-based physiotherapist will be organised by the screening physiotherapist during the phone call where participants are informed of their trial arm allocation. Participants will be offered clinic-based care but will not be told they are at high risk of poor outcomes (according to the Keele STarT MSK tool). Clinic-based physiotherapy may include a combination of any advice and education to support self-management (eg, advice to exercise, modify activities, lose weight or take simple pain medications if needed), exercise tailored to patients’ activity goals and level of function, graded activity, graded exposure and manual therapy. The number of appointments offered will be at the discretion of the treating physiotherapist.
Usual care	
All participants	Participants randomised to the usual care group will be offered a course of clinic-based (in-person) physiotherapy as is usually provided at the participating public hospital clinics and an initial appointment commensurate with the usual clinic waiting time. An appointment with a clinic-based physiotherapist will be organised by the screening physiotherapist during the phone call where participants are informed of their trial arm allocation. Participants will be offered clinic-based physiotherapy which may include a combination of any advice and education to support self-management (eg, advice to exercise, modify activities, lose weight or take simple pain medications if needed), exercise tailored to patients’ activity goals and level of function, graded activity, graded exposure and manual therapy. The number of clinic-based appointments will be at the discretion of the treating physiotherapist.
Participants in either group can be referred to a specialist pain clinic or to see a psychologist if the treating physiotherapist believes it would be valuable. All treatment for participants in both trial arms will be conducted within weekday business hours (Monday to Friday from 7.30 a.m. to 4.30 p.m.). Waiting time includes weekends and public holidays when the clinics are closed as it is the normal approach to reporting waiting time in participating clinics.	

### Outcomes

The coprimary outcomes are physical function as assessed by the Patient Specific Functional Scale (PSFS) at 6 months postrandomisation and treatment waiting time (from randomisation to first treatment). PSFS scores at 3 and 12 months are secondary outcomes. Participants completing the PSFS list up to five functional tasks currently limited by their musculoskeletal condition and score each task based on their current level of ability—0 (unable to perform activity) to 10 (able to perform activity at the same level as before the complaint). Scores for each activity will be summed and calculated as an average of the total possible score for the participants (determined by the number of identified activities). This generic measure will be used as patients with a wide range of musculoskeletal pain presentations will be included in the trial, and it compares well with disease-specific measures in terms of reliability and validity.^10 12 13^ Treatment waiting time will be assessed as the number of days from randomisation to the first appointment with a physiotherapist (either telephone, video conference or clinic-based). It will also be expressed as the number of days from the referral date to the first appointment with a physiotherapist to understand treatment waiting times across sites before, during and at the end of the trial recruitment period; this will be a secondary outcome.

Secondary outcomes (eg, pain and quality of life), health resource measures (eg, number of clinic-based appointments and number of telehealth appointments), potential mediators (eg, pain self-efficacy and recovery expectations), process measures (eg, adherence and usability of PT eXercises App) and adverse events are outlined in online supplemental appendix 3.

### Data collection methods

Participants will receive a unique link via email or SMS to complete their surveys directly in REDCap. Participants will receive an email or SMS 2 days prior to each time point, reminding them to complete their respective surveys. Two reminders followed by a phone call will be provided to patients who do not complete their surveys at a particular time point. The treating clinicians may remind participants to complete their surveys during appointments. If requested, paper copies of the surveys will be posted (including a pen and a postage-paid return envelope) to participants with their responses entered directly into REDCap by an assessor blind to group allocation. If required, a study investigator may contact participants to assist them in completing their surveys, so they can post their survey responses to the screening physiotherapist via a postage-paid return envelope.

### Sample size

A total sample of 368 participants will provide 90% power to exclude a noninferiority margin of 0.7 points on the 11-point PSFS with a 15% loss to follow-up,^8^ an SD of 1.9, two-sided α of 5% and PSFS at 6 months in the control arm participants of 5.8—based on a recent trial conducted in the same setting.^14^ A between-group difference of ≤0.7 points will indicate that the new physiotherapy model of care is noninferior (as good as or better) compared with usual clinic-based care. We chose an SD of 1.9 as it is between the mean value of the SD for the PSFS at follow-up in published studies of similar patient populations with the SDs of 1.7,^15^ 2.0^12^ and 2.1^16^. The minimal important difference (MID) for the PSFS ranges from 1.3 (small change) to 2.7 (large change).^17^ Guidelines suggest using a noninferiority margin of 50% (or less preferably) of the expected treatment effect.^18^ Thus, we chose a conservative between-group noninferior margin of 0.7 (50% of the MID of 1.3).

If our physiotherapist-led triage and treatment service is noninferior for improving function at 6 months compared with usual care, we will interpret the findings for treatment waiting time as a coprimary outcome. This hierarchical approach to our coprimary outcomes was selected because consumer feedback suggested that reducing waiting times is not valuable if patient outcomes are adversely affected. Our sample size of 368 participants will provide >80% power to determine a conservative 14-day mean reduction in waiting time (approximately half of the reduction found in the PhysioDirect trial^8^), calculated from medians and interquartile ranges with this formula,^19^ using an SD of 43 (conservative SD from participating public hospital outpatient clinics over the past 5 years), two-sided α of 5% and 15% loss to follow-up.

### Process evaluation: qualitative interviews

A process evaluation, informed by the RE-AIM^20^ framework, will be conducted to better understand the views and experiences of clinicians and patients regarding the two treatment paths in the trial. This information would be useful regardless of the trial result, allowing a better understanding of trial outcomes and assisting in identifying potential barriers and facilitators to future implementation. RE-AIM considers the target population (*Reach* (eg, representativeness), *Effectiveness* of the service, whether clinicians, patients and key stakeholders are willing to *Adopt* the service, fidelity (eg, clinician and patient adherence), barriers and facilitators to *Implementation* and the extent to which the service can become part of practice or policy (Maintenance)). RE-AIM will use quantitative data sources (eg, health resource use, patient adherence to the intervention and clinician fidelity to the protocol) and qualitative data sources (eg, interviews with physiotherapists, patients and key stakeholders). A more detailed outline of the process evaluation will be published a priori. All qualitative aspects of this study will be reported according to the consolidated criteria for reporting qualitative research (COREQ).^21^

We will attempt to interview 10 physiotherapists involved in delivering the physiotherapist-led triage and treatment service, 20 participants from the usual care group and 20 from the physiotherapist-led triage and treatment service (with a similar number of participants from the three treatment subgroups). The anticipated sample size was informed by the concept of information power that considers the study aim, specificity, theory, dialogue and analysis to guide planning for sample size in qualitative research.^22^ The actual sample size may vary based on the saturation of elicited themes. We will attempt to purposively sample physiotherapists involved in delivering the physiotherapist-led rapid triage phone service by age, gender, familiarity with telehealth and years of experience. We will attempt to purposively sample patient participants to achieve diversity in presenting musculoskeletal condition, age, gender, ethnicity, trial arm, treatment subgroup (if in the intervention group) and response to the intervention.

To gain an in-depth understanding of potential barriers to broader implementation, we will also aim to interview 10 physiotherapists who may be interested in this new model of care but were not involved in the trial, 10 patients who dropped out of this trial or chose to not participate, 10 patients who did not attend their appointments and 10 key stakeholders (n=10) (eg, heads of allied health, physiotherapy departments at hospital sites in NSW, representatives from Sydney Health Partners and government-funded insurance authorities), with the anticipated sample size calculated as per earlier.

An experienced researcher and physiotherapist will conduct all qualitative interviews. The role of the interviewer as an active participant in the research process will be critically acknowledged through reflexivity. The interviewer will take field notes to document personal assumptions and biases, and the research team will critically evaluate how their interactions with participants may be shaped by their prior experiences and beliefs through regular reflective practice and peer debriefing.

In this study, the assumed research paradigm is pragmatism as our trial focuses on evaluating the practicality and effectiveness of a physiotherapist-led triage and treatment service in Australian public health physiotherapy clinics. This approach will allow us to make a practice, actionable critique of our new model of care, drawing on the experiences of patients, their health professionals and other key stakeholders.^23^ The paradigm is appropriate for person-centred research as it focuses on applying different research methods, forms and value hierarchies to gain knowledge, asserting that optimal definitions for social problems emanate from the people experiencing them.^23^

### Blinding

Therapists and assessors will not be blinded. The surveys administered during this trial are self-assessments completed by participants who will be blind to the study hypothesis (ie, will not be aware of what the new model of care involves). Blinding participants to the study hypothesis will be achieved by providing participant information sheets that do not mention the study hypothesis and state that the study is comparing two models of care (without specifying which model is the intervention of interest).

### Statistical methods

A detailed Statistical Analysis Plan and Health Economics Analysis Plan (HEAP) including mock tables will be developed before unblinding and shared with the Data Monitoring and Safety Board (DMSB). Prespecified analyses will be programmed using randomly scrambled treatment allocations. Unblinded results will be presented to the study team once all analyses have been programmed and validated.

### Primary analysis

The purpose of our trial is to assess whether the physiotherapist-led triage and treatment service is as good as or better than usual clinic-based care for improving function and better than usual care in terms of reducing treatment waiting time. The between-arm difference in PSFS scores at 6 months postrandomisation is a coprimary outcome and we have prospectively defined a noninferiority margin of −0.7 points for this analysis. This is the maximal compromise on the outcome we are prepared to tolerate (based on guidelines^18^ and consumer feedback) and still consider the physiotherapist-led triage and treatment service to be clinically noninferior to usual clinic-based care. We will test the null hypothesis that the mean difference in PSFS scores (intervention − usual care) is no greater than −0.7 (H0: mean difference ≤−0.7). To declare noninferiority for the new model of care when compared with usual clinic-based care, the 95% CI around the mean difference should be entirely above the noninferiority margin, that is, the lower bound of the 95% CI must be higher than −0.7. A repeated-measure linear mixed model including all postrandomisation PSFS measurements will be used to generate an adjusted mean difference and 95% CI representing the comparison of PSFS scores between the intervention and usual care arms at each time point. The model will be adjusted for the baseline PSFS value (to improve statistical precision), hospital site and treatment subgroup allocation.

The between-arm difference in treatment waiting time is the other coprimary outcome, provided the physiotherapist-led triage and treatment service is as good or better than usual clinic-based care for improving function (hierarchical approach). We have prospectively defined an effect size of 14 days or greater to be clinically important (approximately half the reduction found in the PhysioDirect trial).^8^ This is the difference that our consumers and clinician partners consider meaningful. A generalised linear model will be used to generate an adjusted mean difference and 95% CI representing the comparison of treatment waiting times between the intervention and usual care groups. The model will be adjusted for hospital site and treatment subgroup allocation.

Imputation techniques may be considered if more than 5% of any outcome data are missing, depending on patterns within missing data.

### Secondary analysis

Similar linear models will be used to analyse between-group differences in other continuous outcomes, while logistic regression will be used to estimate the treatment effect for binary outcomes. All analyses of secondary outcomes will be adjusted for baseline values (if applicable), hospital site and treatment subgroup allocation as per the primary analysis.

### Cost-effectiveness analysis

Costs will be measured using a combination of trial records, administratively linked data, healthcare diaries and published data: healthcare costs (based on local costing models) and intervention costs (clinician time and wage, and other resources required to deliver the interventions). Comparisons made between the new model of care and usual care arms will be generated through the economic evaluation which will estimate the difference in the cost and benefits between the arms of the trial. The economic assessment method will adhere to best practice guidelines for economic evaluations alongside noninferiority trials.^24^

Wherever possible, the costs will be standardised to current prices. Quality-adjusted life-years (QALYs) will be generated through the utilisation of the EuroQolEQ-5D-5L questionnaire outcomes which will be compared with the national Australian value set.^25^ To avoid biases associated with complete case analysis, multiple imputation methods may be employed to impute missing data as described in more detail in the HEAP. Incremental cost per QALY gained will demonstrate the results of the economic evaluation.

Predefined equivalence margins for costs and QALYs will be chosen as recommended by guidelines.^24^ Incremental costs and QALYs will be calculated in terms of the difference between those in the physiotherapist-led triage and treatment service group and the usual care group. Bootstrapping will be used to estimate a distribution around costs and QALYs and will be plotted on a cost-effectiveness plane. The cost-effectiveness plane and the predefined equivalence margins will be used to determine the probability of the physiotherapist-led triage and treatment service being noninferior to usual care. One-way sensitivity analyses will be conducted around key cost variables, and a probabilistic sensitivity analysis will be conducted to estimate the uncertainty in all parameters.

### Qualitative interview analysis

All interview data will be analysed using thematic analysis, a method for identifying, analysing and reporting patterns within qualitative data.^26^ We will employ the Braun and Clarke six-step framework for thematic analysis, which includes familiarisation with the data (Step 1), generating initial codes (Step 2), searching for themes (Step 3), reviewing themes (Step 4), defining and naming themes (Step 5) and producing the report (Step 6).^27^ In practice, two researchers will independently familiarise themselves with the interviews (via audio-recording or transcribed interviews using the Otter AI software), record initial observations and identify concepts relevant to the questions asked. The two researchers will then organise concepts into broader themes and subthemes in NVivo that will be discussed with the other co-investigators. Any disagreements in categorising concepts into themes and subthemes will be discussed and resolved. The mapping of themes and subthemes will be iterative as new data emerges. Interview themes and subthemes will be matched to the appropriate five dimensions of the RE-AIM^20^ framework (Reach, Effectiveness, Adoption, Implementation and Maintenance) to further understand contextual factors. Interviews will stop after three consecutive interviews where no new themes have emerged (data saturation).

### Data monitoring

A Data Safety and Monitoring Board (DSMB) charter will be developed to define the roles and responsibilities of an independent DSMB. The independent DSMB will be convened to oversee this trial. The Trial Steering Committee will appoint three members to the DSMB. This will include a member with scientific expertise in the management of musculoskeletal pain in the physiotherapy outpatient department, a member with experience and expertise in clinical trial conduct and methodology and an experienced biostatistician. One of the DSMB members will be the Chair. The Chair will provide DMSB members with a quarterly report that identifies all AEs and SAEs reported during that period. If the quarterly review of AE data does not raise any concerns, the Chair will report this to the other members of the DSMB and to the study team. However, if these reviews suggest any significant risk of harm or unknown or uncertain risks to the trial participants, the Chair will convene a full meeting of the DSMB to review the AE data to make recommendations about whether the trial should continue, continue with modifications or be stopped. Any unintended consequences or AEs will be compared between groups and will be reported at the end of the trial. There will be no interim analyses of outcome data.

### Auditing

Internal audits may be conducted quarterly to carry out data validation for all data entered into the REDCap database manually. A random sample of 10% of all survey data entered into REDCap manually by a member of the research team will be reviewed by a second team member to ensure that data have been entered and recorded accurately. If errors are detected, corrections will be made. In the event of greater than 10% of data being incorrect, a further sample of 10% will be drawn. The new acceptable error rate will then reduce to 9%, and if the observed error rate is greater than the new acceptable error rate then a further 10% sample will be tested (new acceptable error rate of 8%). This process will continue reducing the acceptable error rate by 1% until the error rate drops below the acceptable threshold. Audits will also be conducted to ensure that correct procedures are being followed for patient invitation, screening procedure, eligibility criteria, consent and intervention delivery.

### Access to data

Study data will be stored within The University of Sydney’s secure REDCap server. This web-based software will be managed by a member of the research team. This server is secure, stable and backed up daily in compliance with national, state and district privacy and confidentiality obligations. This database will be username and password-protected and can only be accessed by personnel named on the ethics application. Only the screening physiotherapists will have access to identified data. All other study investigators will only have access to deidentified data. All study data and documents will be retained for a minimum of 15 years after the completion of the study. Following this period, destruction of the data and documents will occur in a secure manner as per the privacy act.

### Consumer involvement

We have engaged with consumers and clinician-researchers from study conception (including when writing our Medical Research Future Fund grant funding application for this project) and have formed a trial Consumer Advisory Committee that will provide ongoing feedback on trial design, conduct and reporting. We have also sought feedback from physiotherapists at our hospital sites, and consumers with lived experience of musculoskeletal pain who have presented to these sites, to ensure that our new model of care is as relevant and acceptable as possible.

### Ethics and dissemination

The Human Research Ethics Committee (RPAH Zone) of the Sydney Local Health District has approved this trial (protocol number X24-0090 and 2024/ETH00585). Approval must be sought from the trial’s Steering Committee and the ethics committee for any amendments prior to implementation.

Findings from this trial will be published in peer-reviewed journals and presented at national and international conferences. There will be no identifiable information related to trial participants in any published report or conference presentation. Deidentified individual data will be made available via consultation with the trial team and the approving ethics committee. Deidentified data will be stored on the University of Sydney’s secure digital data servers as outlined in the trial’s research data management plan. We will offer a plain language summary of the findings from the study to all consenting participants.

## supplementary material

10.1136/bmjopen-2024-091293 online supplemental file 1

10.1136/bmjopen-2024-091293 online supplemental file 2

10.1136/bmjopen-2024-091293 online supplemental file 3

## References

[1] Safiri S, Kolahi A, Cross M (2021). Prevalence, Deaths, and Disability‐Adjusted Life Years Due to Musculoskeletal Disorders for 195 Countries and Territories 1990–2017. *Arthritis & Rheumatology*.

[2] Health AIo Welfare (2022). Australian Burden of Disease Study 2022.

[3] Australian Prudential Regulation Authority Private health insurance membership and coverage 2022. https://www.apra.gov.au/quarterly-private-health-insurance-statistics.

[4] Sydney Local Health District (2024). Audit of waiting times across outpatient physiotherapy departments.

[5] Childs JD, Fritz JM, Wu SS (2015). Implications of early and guideline adherent physical therapy for low back pain on utilization and costs. BMC Health Serv Res.

[6] Liu X, Hanney WJ, Masaracchio M (2018). Immediate Physical Therapy Initiation in Patients With Acute Low Back Pain Is Associated With a Reduction in Downstream Health Care Utilization and Costs. Phys Ther.

[7] Sun E, Moshfegh J, Rishel CA (2018). Association of Early Physical Therapy With Long-term Opioid Use Among Opioid-Naive Patients With Musculoskeletal Pain. JAMA Netw Open.

[8] Salisbury C, Montgomery AA, Hollinghurst S (2013). Effectiveness of PhysioDirect telephone assessment and advice services for patients with musculoskeletal problems: pragmatic randomised controlled trial. BMJ.

[9] Zadro JR, Needs C, Foster NE (2022). Feasibility of delivering and evaluating stratified care integrated with telehealth (‘Rapid Stratified Telehealth’) for patients with low back pain: protocol for a feasibility and pilot randomised controlled trial. BMJ Open.

[10] Hill JC, Garvin S, Chen Y (2020). Stratified primary care versus non-stratified care for musculoskeletal pain: findings from the STarT MSK feasibility and pilot cluster randomized controlled trial. BMC Fam Pract.

[11] Gamble AR, Maher CG, McKay MJ (2024 (under review)). The development of a model of care to reduce waiting times in outpatient musculoskeletal clinics: a mixed-methods study.

[12] Horn KK, Jennings S, Richardson G (2012). The Patient-Specific Functional Scale: Psychometrics, Clinimetrics, and Application as a Clinical Outcome Measure. *Journal of Orthopaedic & Sports Physical Therapy*.

[13] Sterling M, Brentnall D (2007). Patient Specific Functional Scale. Aust J Physiother.

[14] Withers HG, Glinsky JV, Chu J (2024). Remotely delivered physiotherapy is as effective as face-to-face physiotherapy for musculoskeletal conditions (REFORM): a randomised trial. J Physiother.

[15] Navarro-Pujalte E, Gacto-Sánchez M, Montilla-Herrador J (2019). Sensitivity to change of mobility measures in musculoskeletal conditions on lower extremities in outpatient rehabilitation settings. Disabil Rehabil.

[16] Abbott JH, Schmitt J (2014). Minimum Important Differences for the Patient-Specific Functional Scale, 4 Region-Specific Outcome Measures, and the Numeric Pain Rating Scale. *Journal of Orthopaedic & Sports Physical Therapy*.

[17] Pathak A, Wilson R, Sharma S (2022). Measurement Properties of the Patient-Specific Functional Scale and Its Current Uses: An Updated Systematic Review of 57 Studies Using COSMIN Guidelines. *Journal of Orthopaedic & Sports Physical Therapy*.

[18] (2006). Committee for medicinal products for human use (CHMP) guideline on the choice of the non‐inferiority margin. Stat Med.

[19] Wan X, Wang W, Liu J (2014). Estimating the sample mean and standard deviation from the sample size, median, range and/or interquartile range. BMC Med Res Methodol.

[20] Gaglio B, Phillips SM, Heurtin-Roberts S (2014). How pragmatic is it? Lessons learned using PRECIS and RE-AIM for determining pragmatic characteristics of research. Implement Sci.

[21] Tong A, Sainsbury P, Craig J (2007). Consolidated criteria for reporting qualitative research (COREQ): a 32-item checklist for interviews and focus groups. Int J Qual Health Care.

[22] Malterud K, Siersma VD, Guassora AD (2016). Sample Size in Qualitative Interview Studies: Guided by Information Power. Qual Health Res.

[23] Allemang B, Sitter K, Dimitropoulos G (2022). Pragmatism as a paradigm for patient-oriented research. Health Expect.

[24] Bosmans JE, de Bruijne MC, van Hout HPJ (2008). Practical guidelines for economic evaluations alongside equivalence trials. Value Health.

[25] Norman R, Mulhern B, Lancsar E (2023). The Use of a Discrete Choice Experiment Including Both Duration and Dead for the Development of an EQ-5D-5L Value Set for Australia. Pharmacoeconomics.

[26] Braun V, Clarke V, Hayfield N (2019). Thematic Analysis.

[27] Braun V, Clarke V (2006). Using thematic analysis in psychology. Qual Res Psychol.

[28] Zadro JR (2024). Clinimetrics: Keele STarT MSK tool. J Physiother.

