# Supplementary material for: Effectiveness of a physiotherapist-led triage and treatment service on WAITing time for adults with musculoskeletal pain referred to Australian public hospital physiotherapy clinics: a protocol for the WAIT-less trial
Source: BMJ Open. 2025 Jan 15;15(1):e091293. doi: 10.1136/bmjopen-2024-091293 (PMC11752015; doi:10.1136/bmjopen-2024-091293)
Supplement: online supplemental file 1 [file bmjopen-15-1-s001.docx]

| 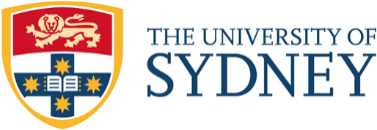 | **School of Public Health** **Faculty of Medicine and Health** |
| --- | --- |
| ABN 15 211 513 464 |  |
| **Dr Joshua Zadro**  *Coordinate Principal Investigator*  *Research Fellow* | Room 10/071  Level 10 North, King George V Building Royal Prince Alfred Hospital  The University of Sydney  NSW 2050 AUSTRALIA  Telephone: +61 2 8627 6782  Facsimile: +61 2 8627 6262  Email: [joshua.zadro@sydney.edu.au](mailto:joshua.zadro@sydney.edu.au)  Web: <http://www.sydney.edu.au> |

**Waiting list trial: PARTICIPANT INFORMATION STATEMENT**

1. **What is this study about?**

You are invited to take part in a research study that will explore a new way of managing the waiting list for people with musculoskeletal conditions referred to the outpatient physiotherapy department. This Participant Information Statement tells you about the study. Knowing what is involved will help you decide if you would like to take part. Please read this study information sheet carefully and ask questions about anything that you do not understand or want to know more about.

Participation in this research study is voluntary.

By giving your consent to take part in this study you are telling us that you:

- Understand what you have read
- Agree to take part in the research study as outlined below
- Agree to the use of your personal information as described

This Participant Information Statement is yours to keep.

Currently, when you are referred to see a physiotherapist at a public hospital outpatient physiotherapy clinic, you are placed on a waiting list. Unfortunately, waiting times for treatment range from 3 to 12 months. Our project will compare a new way of managing the waiting list to the current approach. To find out which is best, half of people will be managed using the new approach and half will be managed using the current approach. We will monitor the two groups for 12 months and compare what happens between the groups. To ensure the groups are similar, the group that you will be placed into will be selected by chance. **There is a 50% chance you will be managed according to the new approach, and a 50% chance you will be managed according to the current approach.** To make the results of our study fair, we will not tell you which group you have been allocated to.

Both ways of managing the waiting list involve common physiotherapy approaches such as in-person appointments, telephone or videoconference appointments, and App-based exercise programs. Therefore, it won't be possible for you to know which group you were allocated to.

If you decide you do not want to participate in the research study, you will be managed according to the current pathway. However, your decision to participate will not affect your current or future relationship with the researchers or anyone else at the University of Sydney or anyone at the participating hospitals. It also won’t affect your position on the waiting list or the quality of care you receive.

1. **Post-study Interview**

After the study, you may be contacted to participate in a phone or online interview to explore your experiences with the physiotherapy you received. The interview will take ~30 minutes and will be audio and/or video recorded. If you do not want a video recording, you will be able to turn off your camera during the interview. Audio from the interview recording will be transcribed and will not contain any details that will identify you.

The interview will be conducted by a researcher from The University of Sydney who was not part of your treating team. The interview will ask you about your experiences, feelings and expectations regarding the physiotherapy you received in this study.

More information about the interviews, including the consent process, will be provided if you are selected for an interview.

1. **Who is running the study?**

This study is funded by the Medical Research Future Fund (MRFF). The study sponsor is The University of Sydney. Neither will benefit commercially from this study.

The people conducting this study are:

- Dr Joshua Zadro, NHMRC Postdoctoral Researcher, Institute for Musculoskeletal Health, University of Sydney and Sydney Local Health District
- Prof Christopher Maher, Director, Institute for Musculoskeletal Health, University of Sydney and Sydney Local Health District
- Mr Andrew Gamble, Physiotherapist, Institute for Musculoskeletal Health, University of Sydney & Sydney Local Health District
- Dr Tarcisio Folly de Campos, Postdoctoral Researcher, The University of Sydney
- Dr Christopher Han, Postdoctoral Researcher, The University of Sydney
- Mr Joshua Hutton, Physiotherapist, Royal Prince Alfred Hospital, Institute for Musculoskeletal Health, University of Sydney & Sydney Local Health District

1. **Who can take part in the study?**

People will be allowed to participate in this study if they:

- are a new referral to the outpatient physiotherapy clinic at any of the hospitals involved in the trial
- have a musculoskeletal condition (e.g. back, neck, shoulder, or knee pain) that would usually be managed by a physiotherapist
- are 18 years or over
- willing to participant and provide follow up data

1. **What does the study involve?**

If you agree to participate in our study, we will send you a survey asking questions about you and your musculoskeletal condition. We kindly ask you to complete these questionnaires and return them back to us via mail (return-paid envelope provided), or by completing the questionnaire online (email or SMS link). After this, you will be randomly allocated (i.e. by chance) to be managed using the new or current approach to managing the waiting list. We will send you another questionnaire at 4-weeks, 3-months, 6-months, and 12-months after joining the study to see how your musculoskeletal condition has changed. These questionnaires will contain similar questions to the first one you will complete. If you desire any more information at any point of the study, relevant contact details will be provided.

After 6 months, we may contact you to participate in a semi-structured one-on-one interview if you agree to participate. This interview may be conducted via telephone or videoconference (e.g. Zoom) or in person at the Institute for Musculoskeletal Health, Level 10 King George V Building, Royal Prince Alfred Hospital. The interview will explore your opinions on the care you received during the study. You will be sent more information about this interview before you decide if you would like to participate.

1. **How much of my time will the study take?**

If you decide to participate in this study, the duration of your treatment is unlikely to be different than if you did not participate in this study. However, we will ask you to complete one survey when you enter the study, and another at 4-weeks, 3-months, 6-months, and 12-months. Each survey will take between 10-15 minutes. You may also be asked to participate in a 30-minute one-on-one interview after the study, but participation is voluntary.

1. **Do I have to be in the study? Can I withdraw from the study once I've started?**

Participation in this study is entirely voluntary. You are not obliged to participate. If you do participate, you can withdraw at any time without having to give any reason and without any penalty. Whatever your decision, it will not affect your relationship with the Hospital, Local Health District and The University of Sydney, or the standard of care you receive now or in the future.

1. **Are there any risks or costs associated with being in the study?**

Aside from giving up your time to complete 5 x 10-15 minutes surveys over 12 months (plus a possible 30 minutes for an interview if you’re interested), we do not expect that there will be any risks or costs associated with taking part in this study.

1. **Are there any benefits associated with being in the study?**

If you are allocated to receive the new approach to managing the waiting list, you may benefit from faster access to physiotherapy care. You may also improve faster because you get treatment sooner. If you are allocated to receive the current approach to managing the waiting list, you will receive the same treatment within the same timeframe as if you had not taken part in the study.

By participating you will be contributing to important research that helps us understand whether our new approach to managing the waiting list is potentially beneficial for people with musculoskeletal conditions. The results will help us develop better ways to improve the quality of care provided to patients in the future.

1. **What will happen to information about me that is collected during the study?**

All data collected will be entered electronically and stored on a research database named REDCap (Research Electronic Data Capture). This is a secure, web-based, non-commercial, data management tool designed for research purposes, hosted and backed up on the University of Sydney servers on a daily basis. No personnel other than the researchers will have access to the research documents. The data will be analysed by the researchers at the University of Sydney. All data for use in journal publications and presentations will be de-identified. The files will be retained for 15 years from the day the study is completed. Once this retention expires, the files will be disposed of using the respective hospital site confidential waste disposal service.

The data may be used for future research purposes; however, Human Research Ethics Committee (HREC) approval will be sought prior to any future use of the data. It will not be shared with local or international collaborators.

1. **Will I be told the results of the study?**

You have a right to receive feedback about the overall results of this study. You can tell us that you wish to receive feedback by ticking a box and leaving your email when you complete the consent form. This feedback will be in the form of a one-page lay summary of the results. You will receive this feedback after the study is finished.

1. **What do I do next?**

When you have read this information, please store it in a safe place. If you understand what you have read and would like to participate, please complete the electronic consent form or sign and return the consent form if corresponding by mail.

If you would like to know more about the study at any stage and ask questions, please feel free to contact Mr Joshua Hutton (PhD candidate and investigator) at [waitinglist.trial@sydney.edu.au](mailto:waitinglist.trial@sydney.edu.au) or (02) 9515 9853.

1. **What if I have a complaint or any concerns about the study?**

This study has been approved by the Ethics Review Committee (RPAH Zone) of the Sydney Local Health District (protocol number: X..-….).

If you have any complaints or concerns about any aspect of this study, you should call our research team who will do their best to address any issues. If your concerns are not able to be addressed, you can contact the Executive Officer of the Ethics Review Committee on (02) 9515 6766 and quote protocol number X..-…..

1. **Complaints and compensation**

If you suffer any injuries or complications as a result of the research project, you will be advised to contact the study team and will be assisted with arranging appropriate medical treatment. If you are eligible for Medicare, you can receive any medical treatment required to treat the injury or complication, free of charge, as a public patient in any Australian public hospital.

In addition, you may have a right to take legal action to obtain compensation for any injuries or complications resulting from the study. Compensation may be available if your injury or complication is sufficiently serious and is caused by unsafe drugs or equipment, or by the negligence of one of the parties involved in the study (for example, the researcher, the hospital, or the treating doctor). You do not give up any legal rights to compensation by participating in this study.

This information sheet is for you to keep.
