# Supplementary material for: Effectiveness of a physiotherapist-led triage and treatment service on WAITing time for adults with musculoskeletal pain referred to Australian public hospital physiotherapy clinics: a protocol for the WAIT-less trial
Source: BMJ Open. 2025 Jan 15;15(1):e091293. doi: 10.1136/bmjopen-2024-091293 (PMC11752015; doi:10.1136/bmjopen-2024-091293)
Supplement: online supplemental file 2 [file bmjopen-15-1-s002.docx]

| in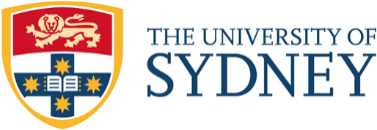 | **School of Public Health** **Faculty of Medicine and Health** |
| --- | --- |
| ABN 15 211 513 464 |  |
| **Dr Joshua Zadro**  *Coordinate Principal Investigator*  *Research Fellow* | Room 10/071  Level 10 North, King George V Building Royal Prince Alfred Hospital  The University of Sydney  NSW 2050 AUSTRALIA  Telephone: +61 2 8627 6782  Facsimile: +61 2 8627 6262  Email: [joshua.zadro@sydney.edu.au](mailto:joshua.zadro@sydney.edu.au)  Web: <http://www.sydney.edu.au> |

**Waiting list trial: PARTICIPANT CONSENT FORM**

I _____________________________________________________________________ *[full name]*

Of _____________________________________________________________________ *[address]*

have read and understood the Participant Information Sheet on the above-named research study

and have discussed the study with ___________________________________________________ [investigator responsible for conducting informed consent].

- I have been made aware of the procedures involved in the study, including any known or expected inconvenience, risk, discomfort, or potential side effect and of their implications as far as they are currently known by the researchers.
- I understand that my de-identified data may be used for future research, and I agree to this.
- I would like to receive a copy of the study results when they become available. My email address

  is: __________________________________________________________________________
- I understand that, during the course of this study, my medical records may be accessed by Sydney Local Health District by regulatory authorities or by the Ethics Committee approving the research in order to verify results and determine that the study is being carried out correctly.
- I understand that the University of Sydney software license for REDCap (Research Electronic Data Capture) will be used to manage the collection and storage of my research data.
- I understand that I may be invited to participate in a post-study interview.
- I have had an opportunity to ask questions and I am satisfied with the answers I have received.
- I freely choose to participate in this study and understand that I can withdraw at any time.
- I consent to the future use of any data I provide for research purposes. I understand that before the researchers can use any data I provide; they must seek additional ethics approval. YES/ NO
- I also understand that the research study is strictly confidential.
- I hereby agree to participate in this research study.
- I consent to the storage and use of my information collected from me for use, as described in the relevant section of the Participant Information Sheet, for:

- This specific research project

- Other research that is closely related to this research project

- Any future research

| Participant Name: ____________________________________________________________________ | |
| --- | --- |
| Participant Signature: _________________________________________________________________ | |
| Date: _____ / _____ / _________  Name of Person conducting informed consent: _____________________________________________ | |
| Signature of Person conducting informed consent: __________________________________________ | |
| Date: _____ / _____ / _________ |  |
| Name of witness to consent form: _______________________________________________________ | |
| Signature of witness to informed consent: _________________________________________________ | |
| Date: _____ / _____ / _________ | |
