# Supplementary material for: Effectiveness of a physiotherapist-led triage and treatment service on WAITing time for adults with musculoskeletal pain referred to Australian public hospital physiotherapy clinics: a protocol for the WAIT-less trial
Source: BMJ Open. 2025 Jan 15;15(1):e091293. doi: 10.1136/bmjopen-2024-091293 (PMC11752015; doi:10.1136/bmjopen-2024-091293)
Supplement: online supplemental file 3 [file bmjopen-15-1-s003.docx]

**Appendix 3:** Table of Outcome Measures

| **Domain** | **Data collection instrument** | **Time points** | | | | | |
| --- | --- | --- | --- | --- | --- | --- | --- |
|  |  | BL | 1^st^ rx | 4  wk | 3  mo | 6  mo | 12  mo |
| **Descriptive data** | | | | | | | |
| Age, DOB, gender, weight, height, BMI, postcode, language spoken at home, country of birth, ethnicity | Baseline assessment questionnaire | X |  |  |  |  |  |
| Education, employment status | Baseline assessment questionnaire | X |  |  |  |  |  |
| Musculoskeletal pain presentation/diagnosis | Completed by screening physiotherapist | X |  |  |  |  |  |
| Medical Record Number (MRN) | Completed by screening physiotherapist | X |  |  |  |  |  |
| Facility (hospital) | Completed by screening physiotherapist | X |  |  |  |  |  |
| Duration of symptoms | Baseline assessment questionnaire | X |  |  |  |  |  |
| Pain location | Baseline assessment questionnaire |  |  |  |  |  |  |
| Pain in other areas | Baseline assessment questionnaire | X |  |  |  |  |  |
| Previous history of sick leave due to complaint | Baseline assessment questionnaire | X |  |  |  |  |  |
| Co-morbidities – is this presence (YES/NO) or number of, or severity of? | Baseline assessment questionnaire | X |  |  |  |  |  |
| Potential non-progressive radiculopathy (if presenting with a condition affecting the lumbar or cervical spine) | Baseline assessment questionnaire | X |  |  |  |  |  |
| **Primary outcomes** | | | | | | | |
| Physical function* | PSFS - 11-point NRS (0= unable to perform activity, 10= able to perform activity at the same level as prior to the presenting health condition). 3-5 activities are nominated by participant | X |  |  | X | X | X |
| Waiting time | This outcome will be assessed as the number of days from randomisation to the first appointment with a physiotherapist (either telephone, video conference or clinic-based). It will also be expressed as the number of days from referral date to the first appointment with a physiotherapist to understand waiting times across sites before, during, and at the end of the trial recruitment period. |  | X |  |  |  |  |
| **Secondary outcomes** | | | | | | | |
| Average pain over the last 24 hours | 11-point NRS (0= No pain at all, 10= worst pain imaginable) | X | Every 2 weeks following randomisation up to 12-months | | | | |
| Time to recovery | Time in days from randomisation to the first day of 7 consecutive days with <2/10 pain |  | Every 2 weeks following randomisation up to 12-months or until recovery. | | | | |
| Quality of life | EQ-5D-5L | X |  |  | X | X | X |
| Risk of poor outcomes | 10-item Keele STarT MSK Tool (scores 0 to 12; low risk, medium risk, high risk) | X |  |  | X | X | X |
| Satisfaction with care | “Overall, how satisfied are you with the care you received as part of this study?” Scored on a 5-point Likert scale (Very satisfied, Satisfied, Neither satisfied nor dissatisfied, Dissatisfied, and Very dissatisfied). As per the UK PhysioDirect trial^1^. |  |  |  | X | X | X |
| **Health resource use** | | | | | | | |
| Number of clinic-based appointments | The number of clinic-based physiotherapy appointments the patient has received during the trial. |  |  | X | X | X | X |
| Number of telehealth appointments | The number of telehealth physiotherapy appointments the patient has received during the trial. |  |  | X | X | X | X |
| Appointment duration | Assessed via treatment recording forms |  | Collected at every appointment | | | | |
| Did not attend rate | Assessed via treatment recording forms and expressed as average per arm and average per site. |  | Collected at every appointment | | | | |
| Healthcare use | Self-reported use of health services and co-interventions, including imaging tests; MBS data, and hospital admissions & emergency department presentations from administrative datasets (only extracted at the final follow-up). |  |  |  | X | X | X |
| Medication use | Self-reported medication use. PBS data from administrative datasets only extracted at the final follow-up. |  |  |  | X | X | X |
| Costs | Costs involved with participant’s care during the trial. |  |  |  | X | X | X |
| **Potential mediators** | | | | | | | |
| Pain self-efficacy | PSEQ includes 10 items scored on a 7-point Likert scale (0= Not at all confident, 6= Completely confident). A total score is calculated by summing the scores for each of the 10 items, yielding a maximum possible score of 60 |  |  | X |  |  |  |
| Recovery expectations | “How likely do you think that your condition will be fully recovered in 6 months?” 11-point NRS (0= Not at all likely, 10= Very likely). Adapted from studies ^2-5^ |  |  | X |  |  |  |
| Reassurance condition is not serious | “How reassured do you feel that there is no serious condition causing your pain condition?” 11-point NRS (0= Not reassured at all, 10= Completely reassured). Adapted from (Traeger, 2019)^2^ |  |  | X |  |  |  |
| Reassurance activity is safe | “How reassured do you feel that continuing with your daily activities is safe?” 11-point NRS (0= Not reassured at all, 10= Completely reassured) Adapted from (Traeger, 2019)^2^ |  |  | X |  |  |  |
| Reassurance of a good prognosis | “How reassured do you feel your pain will improve over time?” 11-point NRS (0= Not reassured at all, 10= Completely reassured) Adapted from (Traeger, 2019)^2^ |  |  | X |  |  |  |
| Reassurance there are management options | “How reassured do you feel that you have treatment options” 11-point NRS (0= Not reassured at all, 10= Completely reassured) Adapted from (Traeger, 2019)^2^ |  |  | X |  |  |  |
| Anxiety | “How tense or anxious have you felt in the past week?” 11-point NRS (0= Not at all, 10= Extremely) adapted from studies ^3-5^ |  |  | X |  |  |  |
| Fear of movement | “How much fear do you have that your pain would be increased by physical activity?” 11-point NRS –(0= No fear, 10= A great deal of fear)^6^ |  |  | X |  |  |  |
| **Process measures** |  |  |  |  |  |  |  |
| Adherence | Self-reported as average number of days per week performing prescribed exercises over the past month (0 to 7 days). Adherence will also be assessed by participants ticking off completed exercises through the PTX App |  |  |  | X | X | X |
| Usability of PT eXercises App | System Usability Scale (SUS) (add scoring details) |  |  |  |  |  | X |
| Clinician intervention fidelity | Treatment recording form | Recorded after every appointment. | | | | | |
| **Additional outcomes** |  |  |  |  |  |  |  |
| Harms | Adverse events and serious adverse events form |  |  | X | X | X | X |
| Success of blinding | Assessor blinding assessed via allocation guesses (and reasons); then forced choice. No need to do this for outcome and therapist blinding as this type of blinding isn’t possible |  |  |  |  |  |  |
| Abbreviations: BL, Baseline; Rx, treatment; wk, week; mo, month; DOB, Date of Birth; BMI, Body Mass Index; PSFS, Patient Specific Functional Scale; NRS, Numeric Rating Scale; EQ-5D-5L, EuroQol-5 Dimensions 5 Levels; PSEQ, Pain Self-Efficacy Questionnaire.  * PSFS collected at 6-months follow-up is a primary outcome for the trial. | | | | | | | |

References:

1. Chris S, Alan AM, Sandra H, et al. Effectiveness of PhysioDirect telephone assessment and advice services for patients with musculoskeletal problems: pragmatic randomised controlled trial. *BMJ : British Medical Journal* 2013;346:f43. doi: 10.1136/bmj.f43

2. Traeger AC, Lee H, Hübscher M, et al. Effect of Intensive Patient Education vs Placebo Patient Education on Outcomes in Patients With Acute Low Back Pain: A Randomized Clinical Trial. *JAMA Neurology* 2019;76(2):161-69. doi: 10.1001/jamaneurol.2018.3376

3. O'Keeffe M, Ferreira GE, Harris IA, et al. Effect of diagnostic labelling on management intentions for non-specific low back pain: A randomized scenario-based experiment. *European Journal of Pain* 2022;26(7):1532-45. doi: <https://doi.org/10.1002/ejp.1981>

4. Zadro JR, O'Keeffe M, Ferreira GE, et al. Diagnostic Labels for Rotator Cuff Disease Can Increase People's Perceived Need for Shoulder Surgery: An Online Randomized Controlled Trial. *Journal of Orthopaedic & Sports Physical Therapy* 2021;51(8):401-11. doi: 10.2519/jospt.2021.10375

5. Zadro JR, O'Keeffe M, Ferreira GE, et al. Diagnostic labels and advice for rotator cuff disease influence perceived need for shoulder surgery: an online randomised experiment. *J Physiother* 2022;68(4):269-76. doi: 10.1016/j.jphys.2022.09.005 [published Online First: 20221017]

6. Verwoerd AJ, Luijsterburg PA, Timman R, et al. A single question was as predictive of outcome as the Tampa Scale for Kinesiophobia in people with sciatica: an observational study. *J Physiother* 2012;58(4):249-54. doi: 10.1016/s1836-9553(12)70126-1 [published Online First: 2012/11/28]
