# Supplementary material for: Effectiveness of a physiotherapist-led triage and treatment service on WAITing time for adults with musculoskeletal pain referred to Australian public hospital physiotherapy clinics: a protocol for the WAIT-less trial
Source: BMJ Open. 2025 Jan 15;15(1):e091293. doi: 10.1136/bmjopen-2024-091293 (PMC11752015; doi:10.1136/bmjopen-2024-091293)
Supplement: Supplementary file 4 [file bmjopen-2024-091293.reviewer_comments.pdf]

PEER REVIEW HISTORY

BMJ Open publishes all reviews undertaken for accepted manuscripts. Reviewers are asked to complete a checklist review form and are provided with free text boxes to elaborate on their assessment. These free text comments are reproduced below.

ARTICLE DETAILS

Title (Provisional)

Effectiveness of a physiotherapist-led triage and treatment service on WAITing time for adults with musculoskeletal pain referred to Australian public hospital physiotherapy clinics: protocol for the WAIT-less trial.

Authors

Hutton, Joshua Michael; Gamble, Andrew R; Maher, Chris G; de Campos, Tarcisio Folly; Han, Christopher S; Coombs, Danielle; Halliday, Mark; Harvey, Lisa; Foster, Nadine E.; Machado, Gustavo C; Anderson, David B; Billot, Laurent; Richards, Bethan; Swain, Michael; McKay, Marnee Jean; Needs, Christopher; Chu, Jackie; Shaw, Tim J; Lung, Thomas; Harris, Ian A; Zadro, Joshua; Core Authorship Group, WAIT-less Trial

---

VERSION 1 - REVIEW

Reviewer 1

Name Chance-Larsen, Kenneth

Affiliation Western Norway University of Applied Sciences, Department of Health and Functioning

Date 05-Sep-2024

COI I confirm that there are no competing interests to declare.

Thank you for the opportunity to review this protocol. This study proposal has great potential to change and improve the model of care for people with musculoskeletal disorders. The authors deserve credit for the quality and comprehensiveness of the submitted protocol.

Below are some specific suggestions to further strengthen certain aspects of the protocol:

1. Eligibility Criteria: It would be beneficial to clarify if self-referring patients will be considered for inclusion in the trial, if this is a relevant access route to physiotherapy.

2. Clarification on Exclusion Criteria: In Figure 1 on page 10, under 'Screening' and 'Enrolment,' you list 'Other reasons' under 'Excluded.' Can you elaborate on what these 'Other reasons' might include?

3. High-risk patients: For patients randomised to the intervention group and identified as high risk of poor outcome, will these patients be informed about this categorisation (directly or indirectly)? If so, how will the potential detrimental consequences (e.g., fear, anxiety) be addressed?

4. Treatment Waiting Time: Regarding the co-primary outcome of treatment waiting time, can you clarify if the number of days includes days when the clinics are closed (e.g., weekends)? Additionally, it might be useful to clarify whether the clinics operate with the same or similar opening hours to account for patient preferences (e.g., appointment times outside of working hours), which might influence how soon appointments can be offered.

5. Premature analysis?: On page 15 (lines 38-42), you state that a process evaluation "… will be conducted to better understand how and why the physiotherapist-led triage and treatment service was as **good or better than** usual clinic-based care, …". This appears to be a premature analysis of the results.

6. You present a thorough sample size calculation for the RCT, but no rationale is given for the number of participants for the qualitative interviews. It would strengthen the protocol to provide such a rationale, for example via the concept of information power (Malterud et al., 2016).

7. Qualitative study reporting standards: The planned mapping of themes and sub-themes to the RE-AIM framework offers a useful guide to the qualitative analyses. It might also be useful to state if the qualitative study will be reported in accordance with available standards (e.g. O'Brien et al., 2014).

8. Research paradigm: Identification of the assumed research paradigm would be useful.

9. Reflexivity in Qualitative Arm: Acknowledging the importance of reflexivity when describing the role of the qualitative researchers would enhance the outline of the qualitative arm.

Good luck with the progression of the study.

Kind regards,
Ken Chance-Larsen

References

Malterud, K., Siersma, V. D., & Guassora, A. D. (2016). Sample Size in Qualitative Interview Studies: Guided by Information Power. *Qualitative Health Research*, *26*(13), 1753–1760. https://doi.org/10.1177/1049732315617444

O’Brien, B. C., Harris, I. B., Beckman, T. J., Reed, D. A., & Cook, D. A. (2014). Standards for Reporting Qualitative Research: A Synthesis of Recommendations. *Academic Medicine*, *89*(9), 1245–1251. https://doi.org/10.1097/ACM.0000000000000388

**Reviewer**	**2**
**Name**	**Cunningham, Caitriona**
**Affiliation**	**University College Dublin, School of Public Health, Physiotherapy**
**Date**	**16-Sep-2024**
**COI**	**None**

Overall well written proposal for a very worthwhile study -Qualitative analysis section of Methods requires some additional detail.

Minor Revisions:

Is it worth adding 'Musculoskeletal' to keywords

'Telephone new referrals' .... add participants/patients (not just 'referrals' but people)

Eligibility: Has cognitive function been considered? Digital Literacy considered ?

eg. ability to use PhysioTherapy Exercise (PTX) App among cohort who wished to have study pack mailed to them and returned by regular mail - or will this be covered by willingness to participate screening questions at outset ?

Usual Care: Now presumably usual care means potentially waiting up to 12 months (as per introduction) for appointment- does it need to be stated that participants will be offered appointment commensurate with usual wait time or just 'back onto waiting list and no appointment given? 'An appointment with a clinic based physiotherapist will be organised by the screening physiotherapist during the phone call where participants are informed of their trial arm allocation' - If patients given an appointment at earlier stage (even if actual appointment months away), does this really represent usual care? Is there a risk that 'usual care' trial participants get an earlier appointment than would be usual at their clinic site? Perhaps an appointment shouldn't be given in this phone call but participants advised clinic will be in contact - perhaps ethical issues though?

NB process evaluation metrics need to be agreed from outset as may need to collect from Day 1 of trial intervention

Qualitative analysis - some more detail required- light on detail compared to description of quantitative sections eg. give 5 basic steps of Braun and Clarke analysis etc Refer to 'saturation' but consider 'information power' perhaps - also very blunt last line 'Interviews will stop once no new themes are identified (data saturation)'. Themes are identified at

analysis stage rather than during data collection phase. (may become apparent of course whilst collecting but ...)

'The two researchers will develop a framework to organise concepts into broader themes and subthemes in NVivo that will be discussed with the other co-investigators'.? Is a framework analysis approach being proposed?

Understand that plan to Identify themes, subthemes and map to RE-AIM but is another framework proposed ?

Recommend further qualitative researcher review and input for this section with supporting references given.

VERSION 1 - AUTHOR RESPONSE

Reviewer #1

Dear Research Team,
Thank you for the opportunity to review this protocol. This study proposal has great potential to change and improve the model of care for people with musculoskeletal disorders. The authors deserve credit for the quality and comprehensiveness of the submitted protocol. Below are some specific suggestions to further strengthen certain aspects of the protocol.

1. Eligibility Criteria: It would be beneficial to clarify if self-referring patients will be considered for inclusion in the trial, if this is a relevant access route to physiotherapy.

Response: Patients are unable to self-refer to these outpatient physiotherapy departments in the Australian public health system. In response to this comment, we have reviewed and modified the text in the section Eligibility Criteria on page 07 of 25. The new sentence now reads:

> *"Patients referred to participating outpatient physiotherapy clinics are typically referred from Hospital Emergency Departments, inpatient units, outpatient hospital clinics and local General Practices. Occasionally patients can self-refer but only at some hospitals and in extenuating circumstances."*

2. Clarification on Exclusion Criteria: In Figure 1 on page 10, under 'Screening' and 'Enrolment,' you list 'Other reasons' under 'Excluded.' Can you elaborate on what these 'Other reasons' might include?

Response: We have reviewed and removed 'Other reasons' from Figure 1 in the section Eligibility Criteria on page 08 of 25. The new Figure 1 now reads:

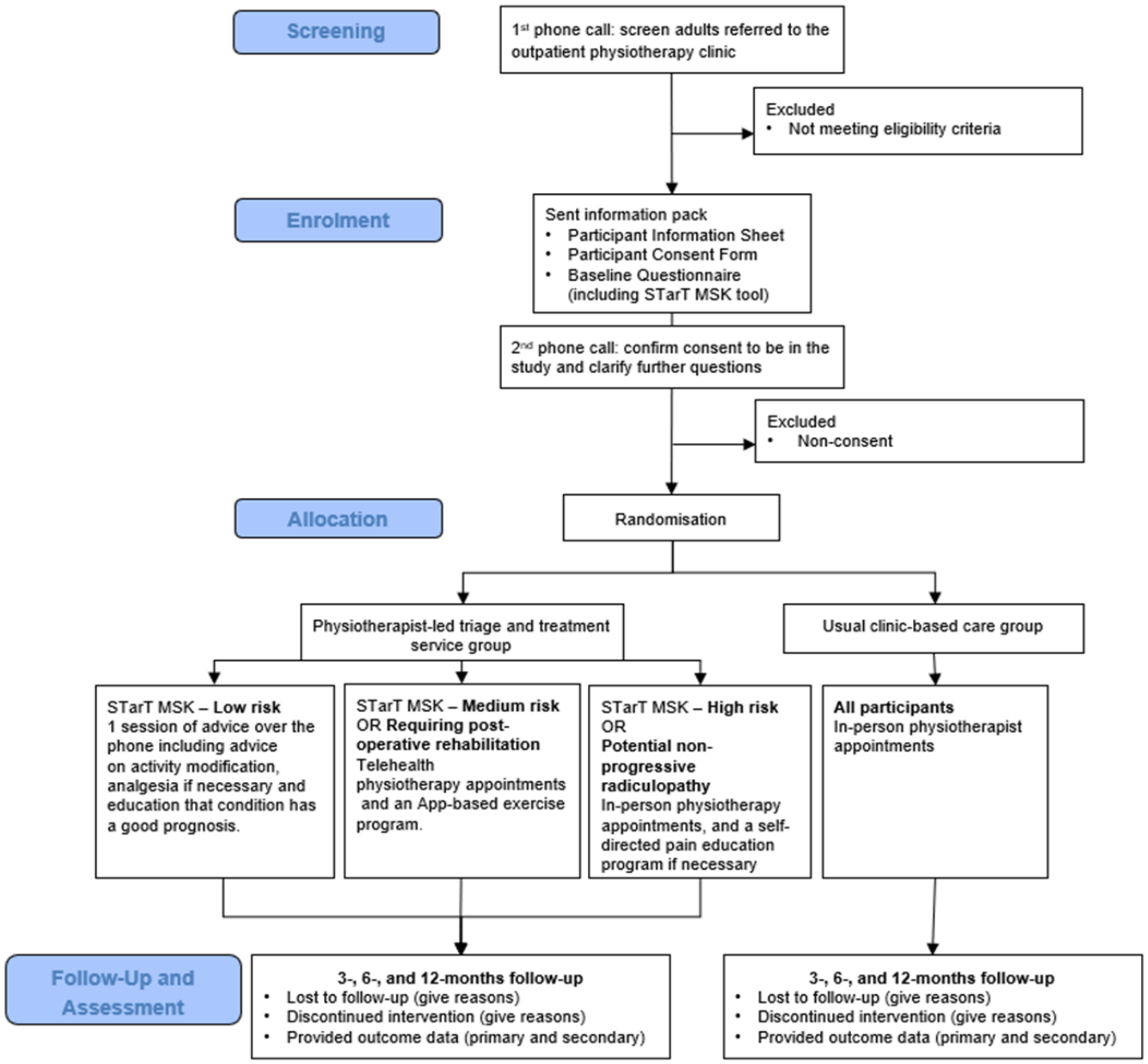

3. High-risk patients: For patients randomised to the intervention group and identified as high risk of poor outcome, will these patients be informed about this categorisation (directly or indirectly)? If so, how will the potential detrimental consequences (e.g., fear, anxiety) be addressed?

Response: Participants categorised in the high-risk sub-group will be offered clinic-based care but will not be told they are at high risk of a poor outcome. We have added this to the manuscript on page 12 of 25, where we address this comment. See below the highlighted sentence:

> *"Participants will be offered clinic-based care but will not be told they are at high risk of poor outcome (according to the Keele STarT MSK tool)."*

4. Treatment Waiting Time: Regarding the co-primary outcome of treatment waiting time, can you clarify if the number of days includes days when the clinics are closed (e.g., weekends)? Additionally, it might be useful to clarify whether the clinics operate with the same or similar

opening hours to account for patient preferences (e.g., appointment times outside of working hours), which might influence how soon appointments can be offered.

Response: We have added a sentence on page 13 of 25 in the final section of table 1. Physiotherapist-led triage and treatment service and usual care protocol. To address this comment, the new sentence now reads:

> *"All treatment for participants in both trial arms will be conducted within weekday business hours (Monday to Friday from 7.30am to 4.30pm). Waiting time includes weekends and public holidays when the clinics are closed as it is the normal approach to reporting waiting time in participating clinics."*

5. Premature analysis?: On page 15 (lines 38-42), you state that a process evaluation "… will be conducted to better understand how and why the physiotherapist-led triage and treatment service was as good or better than usual clinic-based care, …". This appears to be a premature analysis of the results.
Response: To address the reviewer's comment, and to make sure the meaning of this sentence does not appear to be a premature analysis of the results, we have modified the sentence in the section Process evaluation – qualitative interviews on page 15 of 25. The edited sentence now reads:

> *"A process evaluation – informed by the RE-AIM[21] framework - will be conducted to better understand the views and experiences of clinicians and patients regarding the two treatment paths in the trial. This information would be useful regardless of the trial result, allowing better understanding of trial outcomes and assisting to identify potential barriers and facilitators to future implementation."*

6. You present a thorough sample size calculation for the RCT, but no rationale is given for the number of participants for the qualitative interviews. It would strengthen the protocol to provide such a rationale, for example via the concept of information power (Malterud et al., 2016).

Response: We have revised the anticipated sample size for the qualitative component of our study after considering the concept of information power. See below excerpt from the manuscript:

> *"The anticipated sample size was informed by the concept of information power which considers the study aim, specificity, theory, dialogue and analysis to guide planning for sample size in qualitative research.[24] The actual sample size may vary based on saturation of elicited themes."*

7. Qualitative study reporting standards: The planned mapping of themes and subthemes to the RE-AIM framework offers a useful guide to the qualitative analyses. It might also be useful to state if the qualitative study will be reported in accordance with available standards (e.g. O'Brien et al., 2014).

Response: We have added information about reporting the qualitative aspect of our study according to the consolidated criteria for reporting qualitative research (COREQ). See the second paragraph on page 16 of 25.

"All qualitative aspects of this study will be reported according to the consolidated criteria for reporting qualitative research (COREQ).[22]"

8. Research paradigm: Identification of the assumed research paradigm would be useful.

Response: Thank you for your suggestion to include the identification of the assumed research paradigm. We have now included a new paragraph in the section Process evaluation – qualitative interviews on page 16-17 of 25. The included text change reads:

"In this study, the assumed research paradigm is pragmatism, as our trial focuses on evaluating the practicality and effectiveness of a physiotherapist-led triage and treatment service in Australian public health physiotherapy clinics. This approach will allow us to make a practice, actionable critique of our new model of care, drawing on the experiences of patients, their health professionals and other key stakeholders.[24] The paradigm is appropriate for person-centred research as it focuses on applying different research methods, forms and value hierarchies to gain knowledge, asserting that optimal definitions for social problems emanate from the people experiencing them. [24]"

9. Reflexivity in Qualitative Arm: Acknowledging the importance of reflexivity when describing the role of the qualitative researchers would enhance the outline of the qualitative arm.

Response: We have now added some information about reflexivity when describing the role of the qualitative researchers in this study. The following has been added to the protocol manuscript on page 16 of 25:

"An experienced researcher and physiotherapist will conduct all qualitative interviews. The role of the interviewer as an active participant in the research process is critically acknowledged through reflexivity. The interviewer will take field notes to document personal assumptions and biases, and the research team will critically evaluate how their interactions with participants may be shaped by their prior experiences and beliefs through regular reflective practice and peer debriefing."

Reviewer #2

Overall, well written proposal for a very worthwhile study -Qualitative analysis section of Methods requires some additional detail.

Minor Revisions:

1. Is it worth adding 'Musculoskeletal' to keywords.

Response: Thank you for the suggestion, we have added 'Musculoskeletal' to the keywords.

2. 'Telephone new referrals'.... add participants/patients (not just 'referrals' but people).

Response: To address this feedback, we have altered the manuscript to read the following:

> *"A physiotherapist will telephone participants in the intervention group and match them to different modes and types of care based on baseline characteristics."*

3. Eligibility: Has cognitive function been considered? Digital Literacy considered? (e.g., ability to use PhysioTherapy Exercise (PTX) App among cohort who wished to have study pack mailed to them and returned by regular mail - or will this be covered by willingness to participate screening questions at outset?

Response: We recently evaluated the effectiveness of remotely delivered physiotherapy using the PhysioTherapy Exercise (PTX) App in a randomised controlled trial across five diverse public hospitals in Sydney, Australia (https://www.sciencedirect.com/science/article/pii/S1836955324000171#appsec1). Most participants in this trial were able to use the PTX App without issue, although a very small number needed exercises to be emailed or mailed to them which we will also offer in this trial. Furthermore, since the PTX App is a small part of the WAIT-less model of care and we are conducting a pragmatic trial, participants deciding not to use the App is not an issue; rather, this is something we are interested in understanding more through our adherence measures and interviews.

4. Usual Care: Now presumably usual care means potentially waiting up to 12 months (as per introduction) for appointment- does it need to be stated that participants will be offered appointment commensurate with usual wait time or just 'back onto waiting list and no appointment given? 'An appointment with a clinic-based physiotherapist will be organised by the screening physiotherapist during the phone call where participants are informed of their trial arm allocation' - If patients given an appointment at earlier stage (even if actual appointment months away), does this really represent usual care? Is there a risk that 'usual care' trial participants get an earlier appointment than would be usual at their clinic site? Perhaps an appointment shouldn't be given in this phone call, but participants advised clinic will be in contact - perhaps ethical issues though?

Response: Thank you for this question. Usually when a patient is referred to a public hospital physiotherapy clinic, an administrative staff member books them for the next available appointment. In this trial, the screening physiotherapist will do the bookings instead, and usual care participants will be offered an appointment commensurate with the usual wait time. We have now added this to the manuscript in Table 1. Page 13 of 25.

> *"Participants randomised to the usual care group will be offered a course of clinic-based (in-person) physiotherapy as is usually provided at the participating public hospital clinics and an initial appointment commensurate with the usual clinic wait time."*

5. NB process evaluation metrics need to be agreed from outset as may need to collect from

Day 1 of trial intervention

Response: All process evaluation metrics have been agreed on and are mentioned in this protocol. See health resource and process measures in Appendix 3.

6. Qualitative analysis - some more detail required- light on detail compared to description of quantitative sections e.g. give 5 basic steps of Braun and Clarke analysis etc Refer to 'saturation' but consider 'information power' perhaps - also very blunt last line 'Interviews will stop once no new themes are identified (data saturation)'. Themes are identified at analysis stage rather than during data collection phase. (may become apparent of course whilst collecting but ...)

Response: We have added detail to the qualitative analysis section as also suggested by Reviewer #1. For example, we have included information on information power to support our anticipated sample size, re-worded the section on stopping interviews and added information about the Braun and Clarke 6 step framework.

> *"The anticipated sample size was informed by the concept of information power which considers the study aim, specificity, theory, dialogue and analysis to guide planning for sample size in qualitative research.[23] The actual sample size may vary based on saturation of elicited themes." (Page 16 of 25)*
>
> *"Interviews will stop after three consecutive interviews where no new themes have emerged (data saturation)." (Page 20 of 25)*
>
> *"All interview data will be analysed using thematic analysis; a method for identifying, analysing, and reporting patterns within qualitative data.[27] We will employ the Braun and Clarke six step framework for thematic analysis, which includes familiarisation with the data (Step 1), generating initial codes (Step 2), searching for themes (Step 3), reviewing themes (Step 4), ,defining and naming themes (Step 5), and producing the report (Step 6).[28] In practice, two researchers will independently familiarise themselves with the interviews (via audio-recording or transcribed interviews using Otter AI software), record initial observations, and identify concepts relevant to the questions asked. The two researchers will then organise concepts into broader themes and subthemes in NVivo that will be discussed with the other co-investigators.*
> *" (Page 19 of 25)*

7. 'The two researchers will develop a framework to organise concepts into broader themes and subthemes in NVivo that will be discussed with the other co-investigators'.? Is a framework analysis approach being proposed?

Response: We plan to use thematic analysis and have modified the analysis section to avoid any confusion.

> *"All interview data will be analysed using thematic analysis; a method for identifying, analysing, and reporting patterns within qualitative data.[27] We will employ the Braun and Clarke six step framework for thematic analysis, which includes familiarisation with the data (Step 1), generating initial codes (Step 2), searching for themes (Step 3), reviewing themes (Step 4), defining and naming themes (Step 5), and producing the report (Step 6).[28] In practice, two researchers will independently familiarise themselves with the interviews (via audio-recording or transcribed interviews using Otter AI software), record*

initial observations, and identify concepts relevant to the questions asked. The two researchers will then organise concepts into broader themes and subthemes in NVivo that will be discussed with the other co-investigators. Any disagreements in categorising concepts into themes and subthemes will be discussed and resolved." (Highlighted change found on top of Page 19 of 25)

8. Understand that plan to Identify themes, subthemes and map to RE-AIM but is another framework proposed?

Response: We only plan to map themes and subthemes to RE-AIM as this is the framework we find most useable and have most experience with (e.g. https://bmjopen.bmj.com/content/12/7/e057790, https://pubmed.ncbi.nlm.nih.gov/39268673/).

9. Recommend further qualitative researcher review and input for this section with supporting references given.

Response: Many of our investigator team have track record in qualitative research, and qualitative research nested in randomised trials (e.g. Prof Nadine Foster has approximately 46 qualitative publications in PubMed; Dr Joshua Zadro, 8 qualitative publications, 4 first/last author and Prof Lisa Harvey also has multiple qualitative publications). We have revised the manuscript to ensure supporting references are used.

VERSION 2 - REVIEW

Reviewer 1

Name Chance-Larsen, Kenneth

Affiliation Western Norway University of Applied Sciences, Department of Health and Functioning

Date 10-Dec-2024

COI

Dear researchers.

Thank you for the detailed and comprehensive responses to my comments and suggestions.

I look forward to reading your results and analyses in due course.

Kind regards, Ken Chance-Larsen
